# Discovery of a Novel Immune Gene Signature with Profound Prognostic Value in Colorectal Cancer: A Model of Cooperativity Disorientation Created in the Process from Development to Cancer

**DOI:** 10.1371/journal.pone.0137171

**Published:** 2015-09-01

**Authors:** Ning An, Xiaoyu Shi, Yueming Zhang, Ning Lv, Lin Feng, Xuebing Di, Naijun Han, Guiqi Wang, Shujun Cheng, Kaitai Zhang

**Affiliations:** 1 State Key Laboratory of Molecular Oncology, Cancer Institute (Hospital), Chinese Academy of Medical Sciences & Peking Union Medical College, Beijing, China; 2 Department of Endoscopy, Cancer Hospital, Chinese Academy of Medical Sciences, Beijing, China; 3 Department of Pathology, Cancer Hospital, Chinese Academy of Medical Sciences, Beijing, China; Peking University Cancer Hospital & Institute, CHINA

## Abstract

Immune response-related genes play a major role in colorectal carcinogenesis by mediating inflammation or immune-surveillance evasion. Although remarkable progress has been made to investigate the underlying mechanism, the understanding of the complicated carcinogenesis process was enormously hindered by large-scale tumor heterogeneity. Development and carcinogenesis share striking similarities in their cellular behavior and underlying molecular mechanisms. The association between embryonic development and carcinogenesis makes embryonic development a viable reference model for studying cancer thereby circumventing the potentially misleading complexity of tumor heterogeneity. Here we proposed that the immune genes, responsible for intra-immune cooperativity disorientation (defined in this study as disruption of developmental expression correlation patterns during carcinogenesis), probably contain untapped prognostic resource of colorectal cancer. In this study, we determined the mRNA expression profile of 137 human biopsy samples, including samples from different stages of human colonic development, colorectal precancerous progression and colorectal cancer samples, among which 60 were also used to generate miRNA expression profile. We originally established Spearman correlation transition model to quantify the cooperativity disorientation associated with the transition from normal to precancerous to cancer tissue, in conjunction with miRNA-mRNA regulatory network and machine learning algorithm to identify genes with prognostic value. Finally, a 12-gene signature was extracted, whose prognostic value was evaluated using Kaplan–Meier survival analysis in five independent datasets. Using the log-rank test, the 12-gene signature was closely related to overall survival in four datasets (GSE17536, n = 177, *p* = 0.0054; GSE17537, n = 55, *p* = 0.0039; GSE39582, n = 562, *p* = 0.13; GSE39084, n = 70, *p* = 0.11), and significantly associated with disease-free survival in four datasets (GSE17536, n = 177, *p* = 0.0018; GSE17537, n = 55, *p* = 0.016; GSE39582, n = 557, *p* = 4.4e-05; GSE14333, n = 226, *p* = 0.032). Cox regression analysis confirmed that the 12-gene signature was an independent factor in predicting colorectal cancer patient’s overall survival (hazard ratio: 1.759; 95% confidence interval: 1.126–2.746; *p* = 0.013], as well as disease-free survival (hazard ratio: 2.116; 95% confidence interval: 1.324–3.380; *p* = 0.002).

## Introduction

Colorectal cancer (CRC) is the third most common cancer in men (746,000 cases, 10.0% of all cancers) and the second in women (614,000 cases, 9.2% of the all cancers) worldwide [1]. Despite significant advances in understanding its molecular mechanism, CRC remains a major cause of cancer mortality [2]. Previous researches suggested large-scale heterogeneity occurred in CRC [3–5], as well as in many other types of cancer [6–8]. Tumor heterogeneity develops through a sequence of events, guided by clonal selection, where genomic instability contributes to generating a diverse cell population that is subject to selection in a micro-environmental or therapeutic context [9]. Therefore, a novel model that shares similarities with cancer in terms of cell-behavioral and molecular attributes, but that is intrinsically more “organized” is urgently needed.

It has been more than 150 years since Rudolf Virchow first proposed that neoplasms arise “in accordance with the same law, which regulates embryonic development” in 1858. The association between embryonic development and carcinogenesis is widely reported. Furthermore, certain key developmental genes are also involved in carcinogenesis through mutational activation [10]. Through developmental animal models, molecular mechanisms of carcinogenesis have been unveiled and a variety of novel cancer-related molecules, pathways and biomarkers identified [11–13]. Embryonic development and carcinogenesis also share many other similarities with respect to cellular behavior, including epithelial-to-mesenchymal transition (EMT) [14], mesenchymal-to-epithelial transition (MET) [15], and immune-surveillance evasion [16]. Taken together, these findings offer convincing evidence that tumor can be viewed as an aberrant organ that has acquired the capacity for indefinite proliferation through accumulated strikes [17], and that the molecular events which deviate tumor cells from the normal developmental path, are probably accountable for cancer initiation and progression.

Pairwise gene expression correlations (using Pearson correlation) are often used to determine associations between genes in transcriptomic studies [18–20]. The pairwise gene expression correlations in development stage manifest the physiological close or distant associations of gene to gene regulation. Our study indicates that the correlations between genes within a given functional group (immune response) show a remarkably compact and synchronized pattern of gene expression that ensures tight regulation of colonic development. For a given gene, the rank order of its correlation with the remaining members of this gene group, representing the biological association topology, was probably disturbed during carcinogenesis (regulatory relations were stepwise switched from physiological to pathological status). We hypothesized that, if one views a tumor as an aberrant developing organ, the culprit genes responsible for disrupting the integrity of this coordinated gene expression correlation pattern and more specifically, disrupting the rank order of the correlation pattern within this particular gene group during carcinogenesis, probably hold profound prognostic information. We defined this concept as “cooperativity disorientation”, and originally constructed a Spearman transition model to quantify cooperativity disorientation that arises during the progression from colonic development to precancerous progression to cancer, rather than simply concentrate on differentially expressed genes by specific phenotypes.

MicroRNAs (miRNAs) are a class of small non-coding RNAs, ~22 nt in length, that regulate gene expression by binding to the 3′-untranslated region (3′-UTR) of target genes leading to degradation or protein translation inhibition of target genes [21]. MiRNAs are predicted to regulate more than 60% of all protein-coding genes in mammals [22], thereby regulating almost every cellular process [23, 24]. We hypothesized that miRNAs play a pivotal role in CRC patient survival and that the downstream targets of these miRNAs may have prognostic value; this strategy was also adopted by Yang et al. [25] who showed that the expression of 219 miRNA-associated genes was associated with a mesenchymal subtype of serous ovarian cancer associated with poor overall survival (OS) [25].

Although the relationship between cooperativity disorientation, embryonic development and carcinogenesis is still not clear, it is plausible that certain miRNA regulated genes, which play important roles in development stage and contribute to cooperativity disorientation during carcinogenesis, might have a substantial impact on cancer transformation. These genes could be promising candidate prognostic biomarkers.

In this study, we focused on immune response-related genes. The immune response and more specifically, inflammation, has a profound influence on carcinogenesis, which can either kill tumor cells, or, in some circumstances, can be mobilized to facilitate carcinogenesis [26]. The importance of the immune response in carcinogenesis prompted us to determine prognostic biomarkers for CRC. Ours is the first study to examine a range of samples, from human colon embryonic development, colorectal precancerous progression, to CRC samples, in order to simulate the trajectory of human colon development and carcinogenesis. The Spearman transition model we proposed here represents the first step in identifying the culprit genes [differentially expressed genes (DEGs) with a new interpretation on the basis of expression correlation pattern] responsible for disrupting the organized correlation pattern among immune-related genes during carcinogenesis. Using microarray technology and bioinformatics analyses, we identified a 12-gene signature with significant prognostic value, which may be clinically relevant in future.

## Materials and Methods

**Fig 1:**
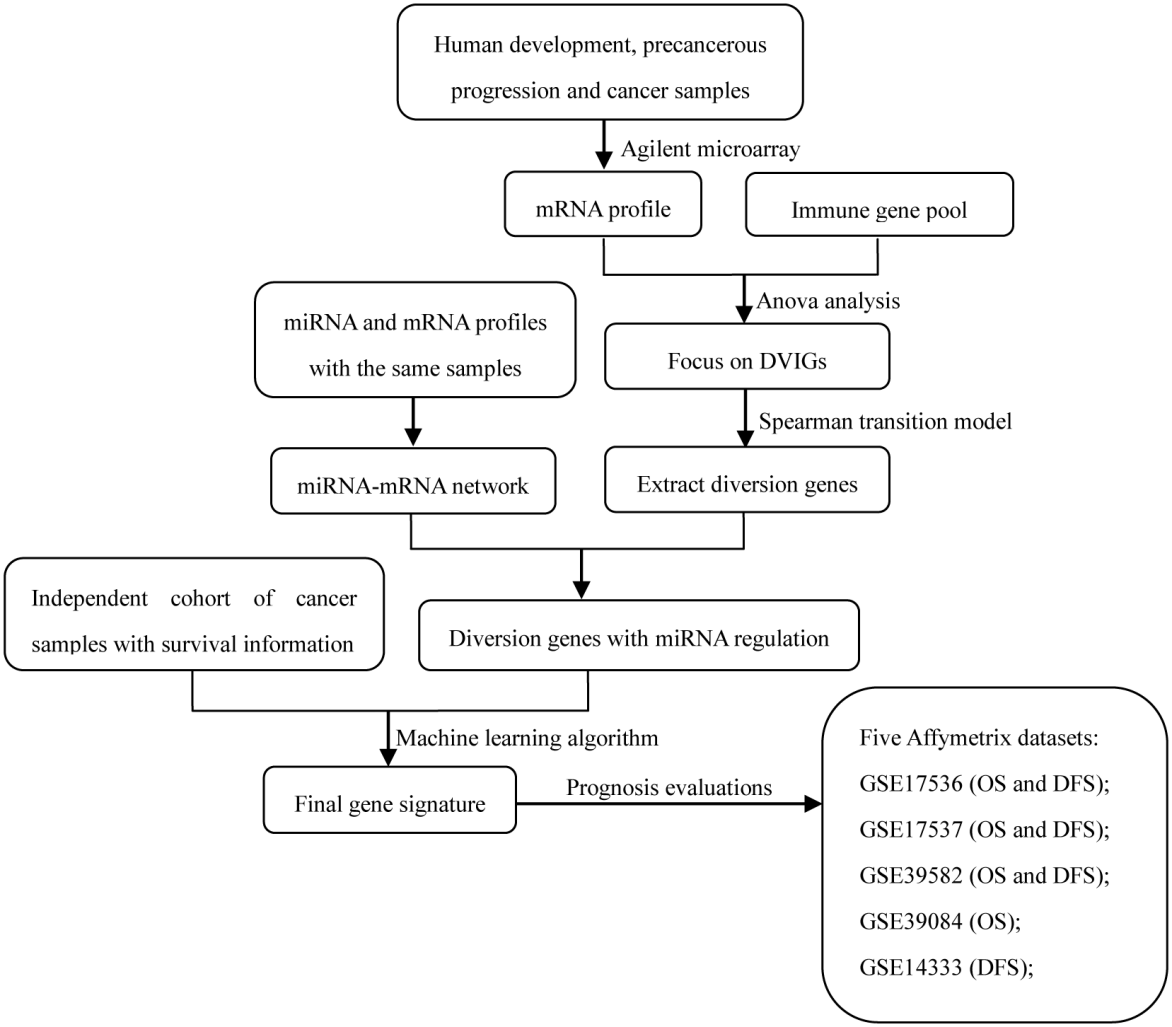
Schematic of the stepwise gene signature selection and evaluation workflow. *CRC* colorectal cancer, *DVIG* development varying immune gene, *OS* overall survival, *DFS* disease-free survival.

A schematic for the study is depicted in Fig 1.

### Patients and samples

In accordance with the principles of gastrointestinal developmental biology [27], developing colon samples were obtained from 20 abortion cases at the Maternal & Child Health Care Hospital of Hai Dian between 2007 and 2009. The samples included whole embryos (WE) at three to five postovulatory weeks (PWs), early embryonic colons (EEC) at eight to ten PWs and middle embryonic colons (MEC) at 14 to 22 PWs. Within 10 minutes of abortion, tissues were rinsed with normal saline, and whole embryos or embryonic colons were carefully separated from fetal tissues with the guidance of a Nikon stereo microscope SMZ1500 (Japan). Embryos/fetuses with known or suspected genetic disorders were excluded.

Normal colorectal mucosal samples were collected from patients with hemorrhoids who received surgical excision in the Department of Colon and Rectal Surgery of Beijing Shi Ji Tan Hospital between 2009 and 2010. Fifty-two CRC samples with OS information were obtained during surgical resection from Zhe Jiang University School of Medicine. Colonoscopy biopsy samples, including colorectal adenomas and adenocarcinomas were obtained from the Department of Endoscopy, Cancer Hospital, Chinese Academy of Medical Sciences, between 2008 and 2011. Patients with the history of familial adenomatous polyposis, hereditary non-polyposis CRC, or inflammatory bowel disease were excluded. Adenoma is defined as dysplasia, carcinoma in situ, suspicion for invasive carcinoma and intramucosal carcinoma; the adenocarcinoma is defined as submucosal invasion by adenocarcinoma [28]. Four to six areas were excised from colorectal neoplasia samples, including the edges and the center of the lesion, according to ASGE guideline [29]. Tissue samples were all snap-frozen in liquid nitrogen immediately after biopsy or surgery and stored at -80°C. A portion of all the samples was subjected to pathological analysis performed by two independent, blinded and experienced pathologists. Samples satisfied with the diagnostic criteria of normal mucosa and neoplasia (neoplastic cells >70%) were enrolled. If more than one biopsy sample was taken from the same patient, these samples were pooled. All donors signed informed consent forms. The use of human tissue samples and the experimental procedures for this study were reviewed and approved by the Ethics Committee of the Cancer Institute and Hospital, Chinese Academy of Medical Sciences.

### RNA isolation

Total RNA was extracted from frozen tissues using TRIzol RNA isolation reagent (Invitrogen, Carlsbad, CA, USA) according to the manufacturer’s specifications. RNA integrity was evaluated using a 2100 Bioanalyzer (Agilent Technologies, Santa Clara, CA, USA). If the RNA integrity number was ≥ 5, the total RNA was further purified using the RNeasy Mini Kit (Cat No.74106, Qiagen, Germany). RNA concentrations were determined with a NanoDrop ND-1000 Spectrophotometer (NanoDrop Technologies, Wilmington, USA).

### Microarray expression profiling and data normalization

After histopathological evaluation and RNA integrity analysis, all the samples were analyzed using Agilent microarrays. Biopsy samples including 6 WE, 6 EEC, 8 MEC, 12 normal, 58 adenoma and 47 adenocarcinoma samples were used for mRNA microarray analysis; of these, 60 samples (2 WE, 6 EEC, 8 MEC, 11 normal, 9 adenoma and 24 adenocarcinoma samples) were also used for miRNA microarray analysis. Purified total RNA samples were labeled and hybridized to Agilent 4×44K Whole Human Genome Oligo Microarrays (G4112F) according to the manufacturer’s instructions. For the miRNA arrays, total RNA was analyzed with an Agilent 8×15K Human miRNA Microarray V3 (G4470C).

The mRNA and miRNA microarray raw data were normalized using the GeneSpring GX software, version 11.5 (Silicon Genetics, Redwood City, CA, USA). For the mRNA expression data, a total of 41,091 single probes were obtained according to GeneSpring’s default setting. The expression value for a particular gene was determined as the median value of all probes mapping to this gene. Eventually, the expression values of 18,986 genes were obtained. Measured miRNAs were deemed present if their signal could be detected in at least 50% of the samples within each sample type. The expression profiles were acquired for 96 miRNAs. The raw and processed data have been deposited in the National Center for Biotechnology Information (NCBI) Gene Expression Omnibus (GEO) database with the series accession numbers GSE71187 (mRNA data) and GSE71130 (miRNA data).

### Affymetrix microarray data collection, preprocessing, and normalization

The raw data for five human colorectal cancer mRNA microarray studies (Table 1) were downloaded from GEO. The combined data set contained a total of 1,094 samples was processed on Affymetrix HG-U133A Plus2 (GPL570) arrays, which contain 52,475 probes. Normalized expression values were obtained via the robust multi-array average (RMA) algorithm and further quantile normalized using the “affy” Bioconductor package. The ComBat algorithm was utilized to eliminate potential batch effects with the Bioconductor package “inSilicoMerging”. The expression levels of 20,184 genes were obtained as the median value of all probes mapping to a particular gene. All clinical information was extracted from the original publications. Among these five datasets, GSE17536, GSE17537 and GSE39582 contain both OS and disease-free survival (DFS) information. GSE39084 contains OS data only, while GSE14333 contains DFS information only.

**Table 1:** Affymetrix microarray datasets included in this study, used to evaluate the prognostic value of our 12-gene signature.

Characteristics	Samples				
	GSE17536	GSE17537	GSE39582	GSE39084	GSE14333
***Number***	177	55	566	70	226
***Year***	2009	2009	2013	2014	2010
***Country***	American	American	France	France	Australia
***Sex***					
Male	96	26	310	35	120
Female	81	29	256	35	106
***Age***					
Mean±SD (years)	65.5±13.1	62.3±14.4	63.0±19.0	59.2±18.3	60.0±13.0
***T status***					
T1+T2	*NR*	*NR*	57	13	*NR*
T3+T4	*NR*	*NR*	486	57	*NR*
***N status***					
N0	*NR*	*NR*	302	35	*NR*
N1	*NR*	*NR*	134	20	*NR*
N2	*NR*	*NR*	104	15	*NR*
***M status***					
M0	*NR*	*NR*	482	48	*NR*
M1	*NR*	*NR*	61	22	*NR*
***AJCC stage***					
Stage I+II	81	19	297	31	*NR*
Stage III+IV	96	36	265	38	*NR*
***Pathologic grade***					
G1	16	8	*NR*	*NR*	*NR*
G2	134	25	*NR*	*NR*	*NR*
G3	27	3	*NR*	*NR*	*NR*
***AdjCTX***					
Yes	*NR*	*NR*	233	*NR*	22
No	*NR*	*NR*	316	*NR*	204

Abbreviations: *SD*, standard deviation; *AdjCTX*, whether chemotherapy was used; *NR*, not reported. Note: GSE39582 has 566 samples, including 562 samples with clear OS information and 557 samples with clear DFS information.

### Identifying “development-varying immune-related genes” (DVIGs)

WE, EEC, MEC and normal samples represented samples at different stages of human colonic development; adenomas were regarded as precancerous lesions; and adenocarcinomas represented the cancer stage. Genes that fell under the Gene Ontology (http://www.geneontology.org) term GO:0006955 were considered immune response-related genes; this resulted in 1028 genes of which 972 were present in our mRNA microarray data. ANOVA was used to retrieve 665 DVIGs that were differentially expressed during the developmental stage (FDR<0.0001).

### Establishment of the Spearman transition model

A detailed description of the Spearman transition model is presented in S1 Methods.

### Construction of a miRNA-mRNA regulatory network

A miRNA-mRNA regulatory network was generated based on sequence algorithms (miRanda [30], TargetScan [31], PicTar [32]) and microarray data (60 biopsy samples with both miRNA and mRNA microarray data). A miRNA-mRNA regulatory pair was regarded as solid only if they satisfied at least two sequence algorithms and if their expression levels were significantly and inversely correlated (FDR<0.01).

### Establishing a CRC gene signature using machine learning applied to mRNA expression profiles of primary clinical samples

Of the 52 surgically excised CRC samples, we selected 19 and 22 cases where patients survived longer (“Good group”) or shorter (“Poor group”) than five years (after surgery) to train a random forests machine learning model. Briefly, genes were ordered by the mean decrease Gini (MDG) criterion, where genes are ranked by their level of influence on the performance of the random forests classification; leave one out cross validation (LOOCV) to estimate “Poor voting” proportion of the test case, which was further treated as predictor in receiver operating characteristic (ROC) test. Genes were then recursively eliminated based on the initial gene ranking, until the area under ROC curve (AUC) was optimized. This algorithm is clearly described in previous research as the AUC-RF algorithm[33].

### Kaplan–Meier survival analysis and Cox regression analysis

Principal component analysis (PCA) was conducted using genes of interest in each downloaded Affymetrix dataset. The first principal component (PC1) captures the greatest amount of total variance in the profiles and was calculated for each patient. Patients were then divided into two groups of equal size based on the rank order of PC1 across their tumor profiles. Kaplan–Meier survival analysis and the log-rank test were used to evaluate the prognostic difference between the two PC1-assigned groups [12]. The Cox proportional hazards regression model was used to evaluate the independence of the prognostic factors in a stepwise manner. Samples in the combined Affymetrix dataset (1,094 samples) with complete information of age, sex, American Joint Committee on Cancer (AJCC) stage (stage), pathological grade (grade) and survival information were used (213 samples for OS Cox analysis and 213 samples for DFS Cox analysis), and a value of *p* < 0.05 was regarded as significant.

### Gene signature validation using random gene sampling

Our strategy was to select a small gene signature with significant prognostic value by narrowing down genes of interest in a stepwise manner. To prove that this method truly predicted survival outcome, n-gene (where the final gene signature contains n genes) random samplings were performed 2000 times in each gene pool. Kaplan–Meier survival analysis was performed with randomly chosen n genes, and the number of times that randomly chosen genes could simultaneously discriminate all the target survival datasets was recorded.

### Statistical Analyses

All statistical analyses were executed using R project software (Version 2.15.1), and Bioconductor (Version 2.11). The R packages “randomForest” (Version 4.6–7) [34] and “pROC” (Version 1.7.1) [35] were used to construct the AUC-RF model. Differentially expressed genes were obtained using the R package “samr”. Kaplan–Meier survival analysis was carried out using R package “survival”. The Bioconductor annotation package “org.Hs.eg.db” (Version 2.8.0) was used to retrieve immune-related genes [36]. Mappings between Affymetrix probes and Entrez gene identifiers were carried out using the Bioconductor package “hgu133plus2.db”. Meta-analysis was conducted with the R package “meta”, and forest plots were made using the R package “rmeta”. Network visualization was performed in Cytoscape (Version 3.2.0) [37].

## Results

### Genes differentially expressed between normal and CRC tissue are significantly enriched for the Reactome term “signaling in immune system”

Differentially expressed genes (DEGs) between normal and CRC tissues were identified using the SAM algorithm (FDR<1e-07). DEGs included 3,226 and 2,538 significantly up- and downregulated genes in CRC. Using Reactome enrichment analysis, conducted with DAVID Bioinformatics Resources 6.7 (http://david.abcc.ncifcrf.gov/), we found that the Reactome term “signaling in immune system” was significantly enriched in CRCs (Bonferroni-adjusted *p* value = 0.004), suggesting a significant association between carcinogenesis and immune-related genes (S1 Table).

### Pearson correlation heatmaps

**Fig 2:**
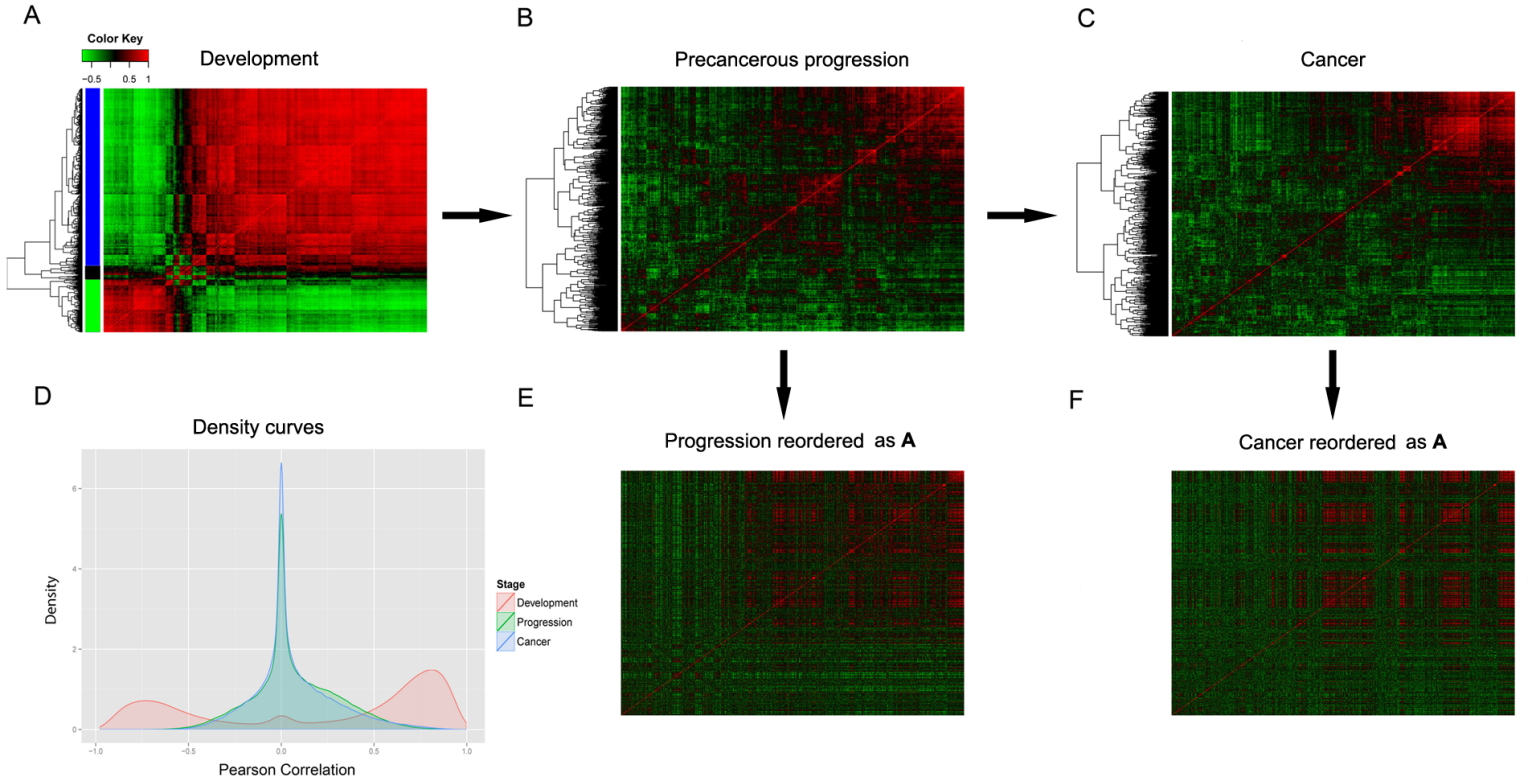
Pearson correlation heatmaps and density curve plot of 665 DVIGs. Heatmaps of adjusted Pearson correlations for 665 DVIGs in **(A)** development, **(B)** precancerous progression and **(C)** cancer, respectively. Genes were clustered into three clusters (highlighted with different colors) by UCA. **(D)** Density plot of pairwise adjusted Pearson correlations for all three stages. The curve for the development stage is bimodal distribution, but unimodal for in progression and cancer stages. In order to render intra-immune vectors comparable, genes were reordered in the progression and cancer stage heatmaps to match the order in the development stage heatmap, to generate **(E)** reordered progression heatmap and **(F)** reordered cancer heatmap. *DVIG*, development varying immune gene; *UCA*, unsupervised clustering algorithm.

Pairwise Pearson correlations among the 665 DVIGs were calculated and adjusted to eliminate bias (adjusted method was described in S1 Methods). Pearson correlation heatmaps (665×665) were constructed for colon samples during colonic development (Fig 2A), progression (Fig 2B) and cancer (Fig 2C) stages. During the developmental stage, three distinct clusters were obtained. Distinct clusters were however not evident during the progression and cancer stages. By superimposing the three Pearson correlation density curves (Fig 2D), a clear bimodal distribution was seen for the developmental stage, in contrast to the unimodal distributions of the progression and cancer stages. Further, the cancer stage had a higher maximum density at a Pearson correlation of zero compared with the progression or developmental stages.

### “Obedient genes” were filtered out using Spearman transition model

**Fig 3:**
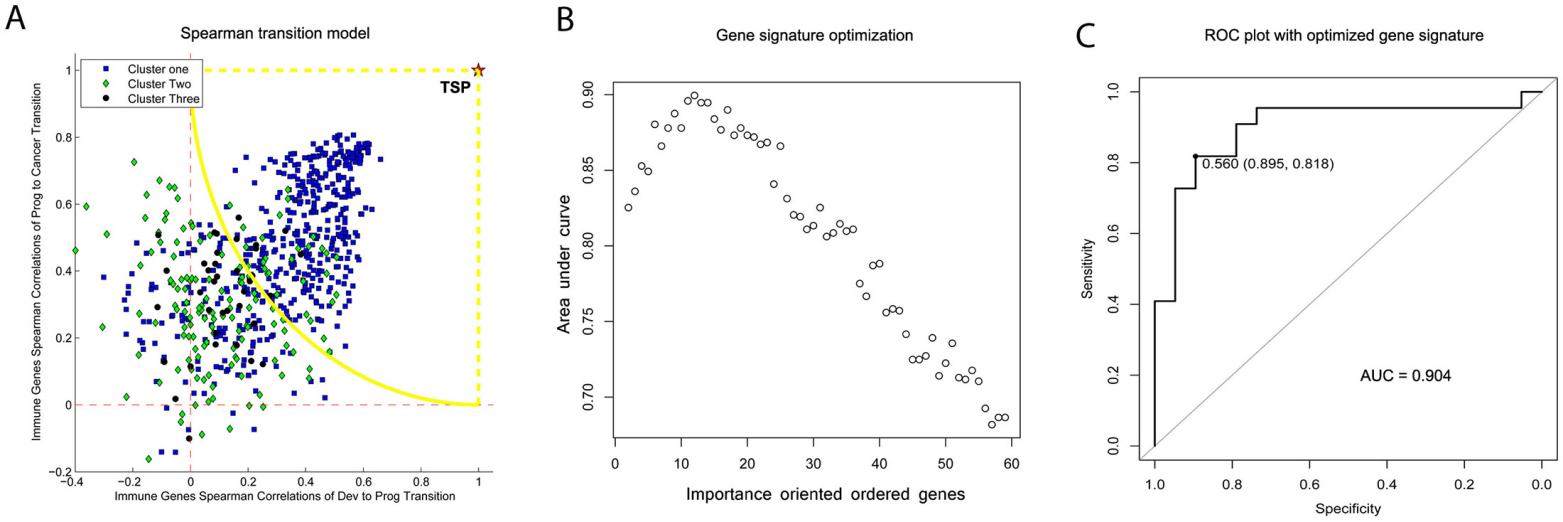
Gene signature optimization based on Spearman correlation transition model and AUC-RF algorithm. **(A)** The 665 DVIGs were projected onto a Spearman correlation transition coordinate system based on their cooperativity disorientation between the consecutive stages. Genes were colored in the same way as in the development heatmap. **(B)** The AUC-RF algorithm was used for gene signature optimization. Genes were recursively removed from an importance-ordered gene list until the largest AUC value was met. **(C)** The biggest AUC of 0.904 (95% *CI*: 0.799~1.000) was obtained when the number of genes were reduced to 12, with 81.8% sensitivity (95% *CI*: 0.636–0.955) and 89.5% specificity (95% *CI*: 0.737–1.000). *Dev*, development; *Prog*, progression; *TPS*, theoretically stable point; *AUC*, area under curve; *DVIG*, development varying immune gene; *CI*, confidence interval.

Pearson correlation heatmaps of DVIGs during the progression and cancer stages were reordered to make all three stages (Fig 2E and 2F, described in S1 Methods). As shown in Fig 3A, the 665 DVIGs were projected onto a Spearman transition coordinate system, with the Spearman transition between development and progression (STD-P, S1 Methods) and between progression and cancer (STP-C, S1 Methods) as the x and y axis coordinates, respectively. Genes were colored in the same way as in the developmental heatmap clustering in Fig 2A. Of the 665 DVIGs, 385 (termed “obedient genes”) fell within the quarter circle’s arc, while the remaining 280 (termed “diversion genes”) fell outside this arc and were used as candidates for downstream selection procedures.

### Diversion genes with one or more miRNA regulators stayed in the gene pool

**Fig 4:**
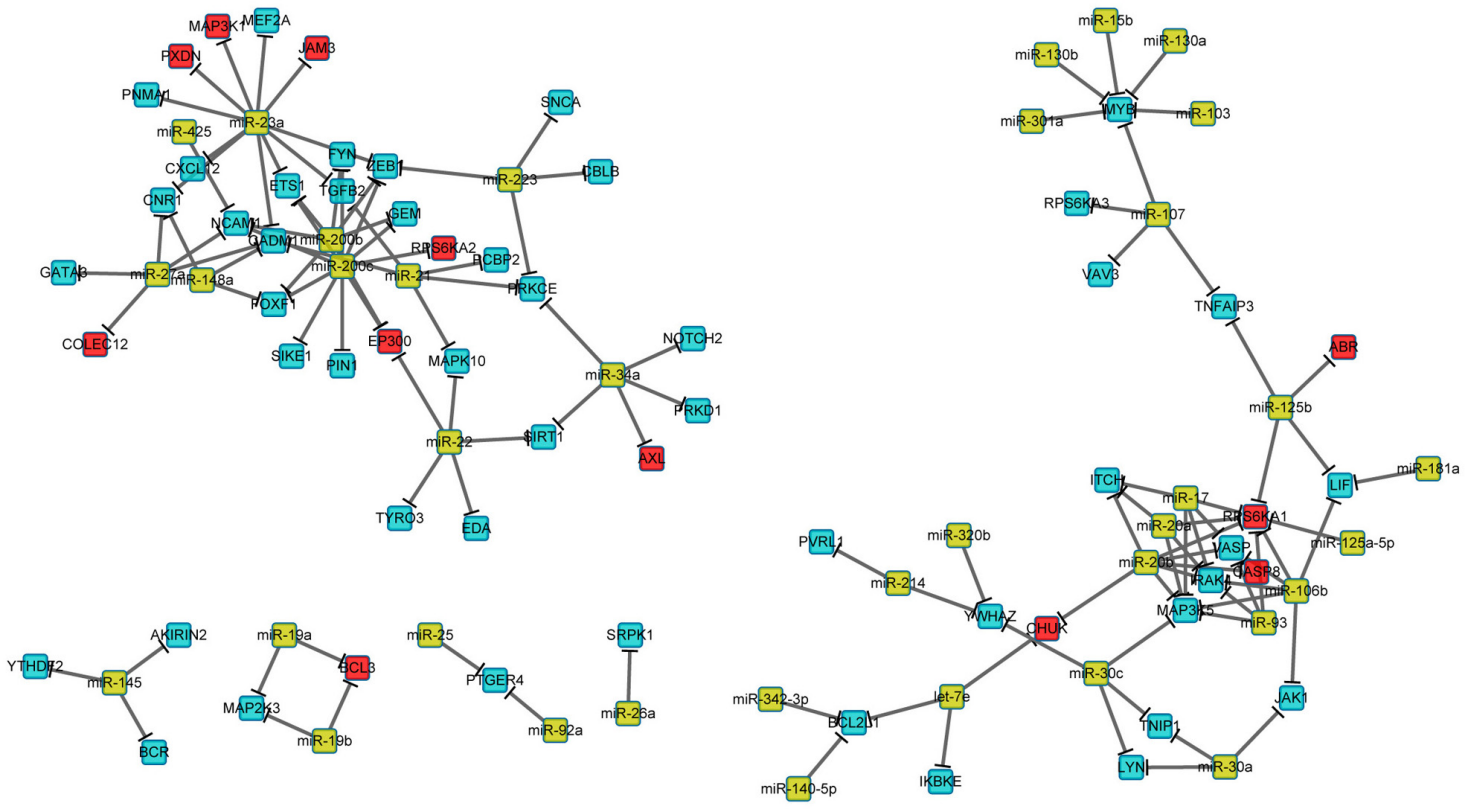
miRNA-mRNA regulatory network. Dark yellow nodes represent miRNAs. Red and sapphire nodes represent mRNAs, among which red ones are genes in the 12-gene signature. Directed solid edges represent miRNA-mRNA regulation.

Using the paired mRNA and miRNA data that were available for 60 of the CRC biopsy samples, we constructed a miRNA-mRNA regulatory network to select diversion genes that had at least one miRNA regulator (S1 Methods). This resulted in 59 diversion genes that were potentially regulated by 37 miRNAs (Fig 4, S2 Table).

### Gene signature optimization by AUC-RF algorithm

The 59 diversion genes were further narrowed down these 59 genes to obtain the subset of genes providing the best prognostic performance. These genes were first ordered according to their importance in discriminating cases by OS (longer or shorter than 5 years) using the random forest algorithm; genes were then recursively eliminated from the bottom of this list until the area under ROC curve (AUC) was optimized (AUC = 0.904, 95% *CI*: 0.799–1.000, Fig 3B). This resulted in an optimized 12-gene signature that had 81.8% sensitivity (95% *CI*: 0.636–0.955) and 89.5% specificity (95% *CI*: 0.737–1.000) in discriminating poor from good OS in 52 surgery samples with a “Poor” voting proportion of 0.560 (Fig 3C). This 12-gene signature is composed of *AXL*, *BCL3*, *COLEC12*, *ABR*, *PXDN*, *EP300*, *JAM3*, *MAP3K1*, *CASP8*, *RPS6KA1*, *CHUK*, and *RPS6KA2*, and is regulated by 16 miRNAs (Fig 4).

### Kaplan–Meier survival and Cox regression analysis confirmed the validity of the 12-gene signature

**Fig 5:**
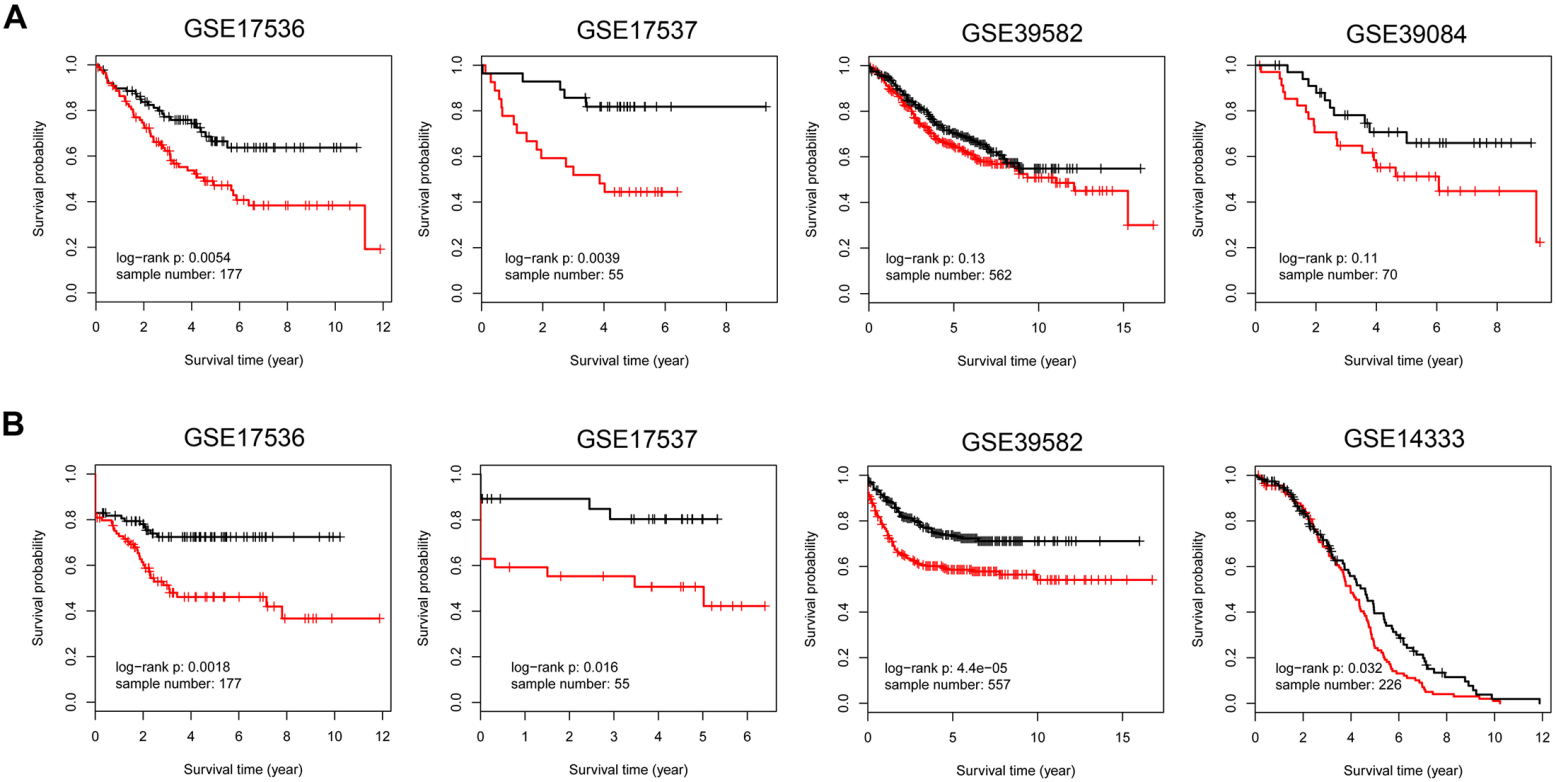
Kaplan–Meier survival analyses and log-rank tests of the 12-gene signature. Kaplan–Meier survival analyses and log-rank tests were conducted to evaluate the prognostic value of the 12-gene signature. (A) The performance of the 12-gene signature in OS discrimination. Datasets with OS information were GSE17536, GSE17537, GSE39582 and GSE39084. (B) The performance of the 12-gene signature in DFS discrimination. Datasets with DFS information were GSE17536, GSE17537, GSE39582 and GSE14333. *OS*, overall survival; *DFS*, disease-free survival.

**Fig 6:**
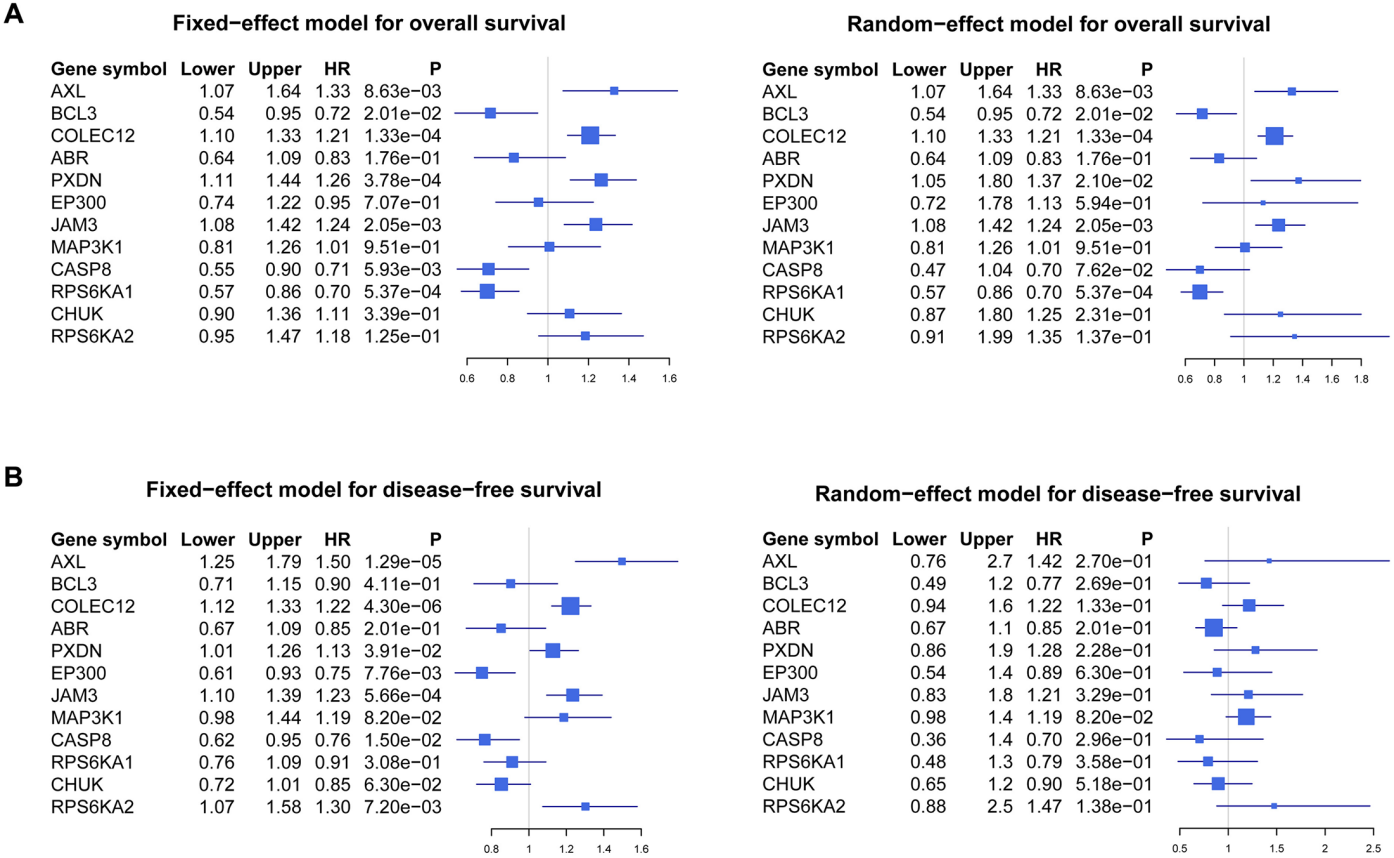
Forest plot of the association between individual genes in the 12-gene signature and CRC survival. (A) Forest plot of the association between individual genes and OS with a fixed-effect model in datasets containing OS information (GSE17536, GSE17537, GSE39582 and GSE39084). Meta-analysis of these 12 genes in four independent datasets was conducted, and *HR*, 95% *CI* of each gene and corresponding *p* value were calculated and plotted in the forest plot. (B) Forest plot of the association between individual genes and DFS with a random-effect model in four datasets containing DFS information (GSE17536, GSE17537, GSE39582 and GSE14333). *CRC*, colorectal cancer; *HR*, hazard ratio; *CI*; confidence interval; *OS*, overall survival; *DFS*, disease-free survival.

Kaplan–Meier survival analysis was conducted to evaluate the prognostic value of the 12-gene signature in five Affymetrix datasets retrieved from the GEO database. The log-rank test results confirmed that the 12-gene signature was closely related to OS in four datasets (Fig 5A; GSE17536, n = 177, *p* = 0.0054; GSE17537, n = 55, *p* = 0.0039; GSE39582, n = 562, *p* = 0.13; GSE39084, n = 70, *p* = 0.11). Furthermore, this 12-gene signature was significantly associated with DFS in four datasets (Fig 5B; GSE17536, n = 177, *p* = 0.0018; GSE17537, n = 55, *p* = 0.016; GSE39582, n = 557, *p* = 4.4e-05; GSE14333, n = 226, *p* = 0.032). Cox regression analysis also confirmed that the 12-gene signature was an independent factor in predicting CRC patient’s OS [Table 2; hazard ratio (*HR*): 1.759; 95% *CI*: 1.126–2.746; *p* = 0.013], as well as DFS (Table 2; *HR*: 2.116; 95% *CI*: 1.324–3.380; *p* = 0.002). Meta-analysis was conducted to evaluate the correlation between each of the 12 genes and survival (OS: GSE17536, GSE17537, GSE39582 and GSE39084; and DFS: GSE17536, GSE17537, GSE39582 and GSE14333) of CRC patients with fixed-effect model (Fig 6A) and random-effect model (Fig 6B).

**Table 2:** Univariate and multivariate analyses of overall survival and disease-free survival (Cox proportional hazards regression model) in 213 patients according to age, sex, stage and the first principal component (PC1) assigned groups calculated with the 12-gene signature.

Factors	Univariate Cox regression		Multivariate Cox regression	
	*HR* (95% *CI*)	*P*	*HR* (95% *CI*)	*P*
***Overall Survival***				
Age	1.013 (0.996~1.030)	0.124	-	-
Sex (Male/Female)	1.049 (0.687~1.601)	0.826	-	-
Stage (I/II/III/IV)	2.505 (1.916~3.275)	**4.491e-13**	2.449 (1.858~3.226)	**1.966e-10**
Grade (I/II/III)	1.915 (1.224~2.997)	**0.005**	1.562 (0.979~2.491)	0.061
PC1*	2.052 (1.316~3.199)	**0.001**	1.759 (1.126~2.746)	**0.013**
***Disease Free Survival***				
Age	0.990 (0.975~1.006)	0.243	-	-
Sex (Male/Female)	0.988 (0.639~1.527)	0.955	-	-
Stage (I/II/III/IV)	4.375 (3.150~6.077)	**<1.000e-15**	4.369 (3.120~6.119)	**<1.000e-15**
Grade (I/II/III)	1.748 (1.110~2.752)	**0.018**	1.179 (0.733~1.894)	0.497
PC1*	2.363 (1.480~3.774)	**1.851e-04**	2.116 (1.324~3.380)	**0.002**

* Based on the rank order of the first principal component (PC1) of the 12 gene signature to divide samples into two groups. Significant *p* values were in bold (*p*<0.05). Abbreviations: *HR*, hazard ratio; *CI*, confidence interval.

### Random gene sampling verified the validity of our methodology

**Fig 7:**
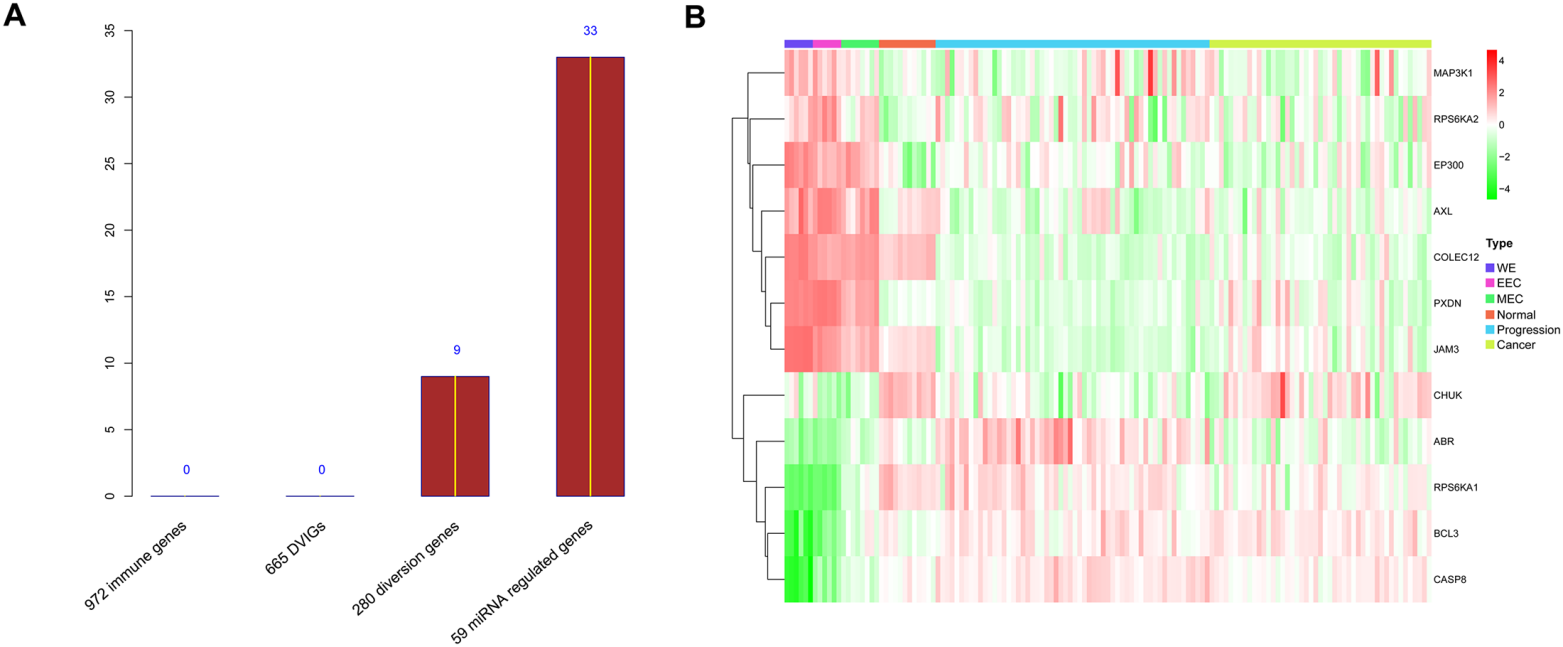
Random gene sampling verified the validity of our step- gene selection procedure. **(A)** Bar plot of the number of times that 12 randomly chosen genes could simultaneously discriminate four survival datasets (OS and DFS in GES17536 and GSE17537, DFS in GSE39582 and GSE14333). (B) Heatmap of 137 biopsy samples established with mRNA expression profile of the 12-gene signature. The mRNA raw data were normalized and then filtered (see “Materials and Methods”). Rows represent genes, and columns represent biopsy samples. Rows, rather than columns, were reordered using UCA, whereas samples of the same type were placed together. *DVIG*, development varying immune gene; *UCA*, unsupervised clustering algorithm; *OS*, overall survival; *DFS*, disease-free survival.

To confirm the validity of our signature selection process, a 12-gene panel was randomly sampled 2000 times across the 972 immune-related genes, 665 DVIGs, 280 diversion genes and 59 miRNA-regulated genes, respectively. The number of times that a randomly chosen 12-gene panel could simultaneously discriminate the survival datasets (OS and DFS in GSE17536 and GSE17537, DFS in GSE39582 and GSE14333), was 0, 0, 9 and 33 for the aforementioned four gene groups, respectively, providing strong evidence for the validity of our hypothesis and gene signature-selection pipeline (Fig 7A).

## Discussion

The intimate association between embryonic development and carcinogenesis makes embryonic development a viable reference model to study cancer, which circumvents the potentially misleading complexity associated with tumor heterogeneity. The molecular resemblances between certain malignant tumors and developing tissues have been reported on the basis of transcription factor activity [38], regulation of chromatin structure [39] and cellular signaling [40]. Important molecules were reported to play a substantial role in both embryonic development and carcinogenesis: *PTCH1* is a key regulator of embryonic development, whose overexpression could drive skin carcinogenesis [41]. Developmental animal models have been used to uncover the complicated molecular mechanisms of carcinogenesis, and a variety of novel and pivotal molecules, pathways and biomarkers have been discovered [12, 42, 43]. For instance, many important signaling pathways, including *NOTCH1* signaling, play a key role in development and in carcinogenesis [44].

These findings prompted scientists to further examine the commonalities between embryonic development and cancer. The two most widely accepted theories are: cancers may, via mutational, epigenetic changes and genome instability, acquire a variety of critical phenotypic traits that compound to facilitate territorial expansion, via proliferative self-renewal, migration and invasion, which are also phenotypic characteristics of embryonic development [45–47]; The second theory is that tumors originate from either tissue stem cells or from their immediate progeny through dysregulation of (normally tightly regulated) self-renewal, so that tumors retain certain key embryonic/stem cell properties [48].

The undeniable relationship between inflammation and cancer has been reported and is firmly established. Infection and chronic inflammation contribute to an estimated 25% of all cancers worldwide [49]. “Smoldering” inflammation refers to chronic, often subclinical inflammation, which has many tumor-promoting effects such as increased proliferation and survival of malignant cells, promotion of angiogenesis and metastasis, subversion of the adaptive immune response, and alteration in the response to hormones and chemotherapeutic agents [50]. Chronic inflammation is particularly relevant to CRC: patients suffering from inflammatory bowel disease (which includes ulcerative colitis and Crohn’s disease), for instance, are at increased risk of developing CRC [51]. Immuno-compromised patients, such as organ transplant recipients [52, 53] or patients with acquired immune deficiency syndrome (AIDS) patients [54, 55], have a remarkable propensity for cancer formation. In addition, certain anti-inflammatory drugs have the ability to reduce CRC risk [56, 57]. In developmental biology, the fetus, which in many ways behaves like an allogenic transplant also evades maternal immune-surveillance by adopting similar mechanisms with tumors [16]. Thus, immune-related genes play a very important role in carcinogenesis as well as in embryonic development.

Ours is the first study to use the mRNA and miRNA expression profiles of human colonic development, colorectal precancerous progression, and cancer samples, to recapitulate the trajectory of human colonic development and carcinogenesis, in an attempt to identify a gene signature with prognostic value. The close association between development and carcinogenesis and the significance of immune-related genes in both processes, substantiates our choice of using immune-related genes as the initial gene pool. The immune-related genes differentially expressed during development are more likely to contain prognostic information of CRC, so we collected 665 DVIGs during the first gene selection step. The correlation pattern within these immune genes was highly organized and synchronized, implying that gene among immune genes were tightly regulated to keep the whole developmental process safe and steady. During carcinogenesis, the culprit genes, which severely sabotage this subtle machinery and drag tumor cell away from normal developmental pathway on the basis of expression correlation, are probably promising indicators of CRC prognosis. In this regard, we initially proposed the Spearman correlation transition model to identify these aberrant genes causing tremendous cooperativity disorientation, and these genes may therefore have significant prognostic value.

In the first step of our Spearman correlation transition model, we calculated pairwise adjusted Pearson correlations for all the 665 DVIGs during development, progression and cancer. Suppose there are two genes A and B, if the correlation value between A and B is close to 1 or -1, the expressions of A and B are strongly synchronized; if the value is close to 0, the expression of A is random to B, and the chance of A and B having a biological association is relatively slim. The expressions of the 665 DVIGs were strongly correlated during colonic development, which implied coordinate regulation of these genes. However, in precancerous progression and cancer stages, the accurate cooperation was corrupted and the expression seems substantially randomized to each other, manifesting massive cooperativity disorientation (a concept we defined in this study) occurred throughout carcinogenesis in terms of immune gene’s expression.

Intra-immune vector (explained in S1 Methods) described the cooperative relation of a particular gene with other immune genes in each stage. A quantitative parameter is needed to describe the shift of this intra-immune relation between consecutive stages for each gene, so we chose Spearman correlation to fulfill the mission. The progression and cancer stage heatmaps were reordered according to the order of the development stage heatmap, to make the development intra-immune vector (DIV), progression intra-immune vector (PIV) and cancer intra-immune vector (CIV) comparable (Fig 2E and 2F). Suppose there is a DVIG named A, for which the STD-P (Spearman correlation between A’s DIV and PIV) is close to 1, indicating in the transition between development and progression stage, the rank order of Gene A’s association with other immune genes was nearly the same; STD-P is close to -1, meaning the rank order was nearly turned upside down; STD-P is close to 0, suggesting the rank order was randomly reshuffled. Likewise, STP-C of Gene A is the parameter denoting the transition of intra-immune cooperativity disorientation between progression and cancer stage. Therefore, we treated STD-P and STP-C as x-axis and y-axis coordinate of their corresponding gene, and in this way, projected all the 665 DVIGs onto the Spearman transition coordinate system.

Point (1, 1) was defined as theoretically stable point (TSP). Suppose a gene was projected onto this TSP, it inferred the cooperativity status (implying biological association) of this gene with other immune-related genes in the whole process of carcinogenesis was absolutely stable. The closer to the TSP a given gene is located in the Spearman correlation transition system, the more stable of this gene during carcinogenesis. Use one Euclidean distance from TSP as the threshold (S1 Methods), we excluded 385 obedient DVIGs and used 280 diversion DVIGs for further narrow-down procedures.

By constructing the miRNA-mRNA regulatory network, we identified 59 (of 280 diversion genes) that were potentially regulated by miRNAs. Using permutation analysis (Fig 7A), our gene signature selection pipeline (Spearman transition model and miRNA-associated genes) proved effective in identifying genes with prognostic significance.

The final 12-gene signature was selected (from the 59 miRNA-regulated genes) using the AUC-RF algorithm (Fig 7B). This 12-gene signature is closely associated with OS of CRC patients, and performed even better in predicting DFS of CRC patients, probably because OS is more likely to be affected by a variety of extraneous factors, such as cardiac diseases, malnutrition etc. Comparing with OS, DFS is much more related to the cancer itself, which might be a sensible explanation for the 12-gene signature (reflecting cooperativity disorientation of the tumor itself) performing better in DFS than OS.

Our results are supported by evidence from the literature. Among the predicted miRNA-mRNA interactions, the regulatory pair *miR-34a*—*AXL* has been implicated in chronic lymphocytic leukemia, breast cancer and other cancer types [58–60]; *miR-200b* and *miR-200c* were proven as major regulators of *EP300*, which suppresses metastasis in ductal adenocarcinomas of the pancreas [61]. Meanwhile, many of the genes in our 12-gene signature are involved in key cancer-related pathways (S3 Table), which have important implications for cancer formation, prognosis and clinical management. *AXL* is an important mediator of inherent and chemotherapy-induced invasion and a predictor of poor clinical outcome in early-stage CRC [60]. Frequent microsatellite instability and consequent loss of *EP300* expression has been reported in gastric and colorectal cancers [62]. *MAP3K1* regulates *JNK* activation and is altered in a variety of cancer types [63]. The 16 upstream miRNAs regulating the 12 genes in the miRNA-mRNA network also influence cancer phenotype and clinical outcome. For example, *miR-23a* antisense enhanced 5-FU-induced apoptosis in CRC cells [64], and was used in a triple miRNA classifier for CRC early detection [65]. Down-regulation of *miR-20b* was observed in various types of CRC, and occurs as an early event of colorectal carcinogenesis in familial adenomatous polyposis tumors [66]. *MiR-17* is a predictive factor for chemotherapy response, a prognostic factor for OS in CRC [67], and also an oncogenic miRNA that regulates tumorigenesis and progression [68].

## Conclusions

As far as we know, this is the first study to use mRNA and miRNA expression profiles of human colonic development, precancerous progression and cancer samples, and together with bioinformatics approaches aimed at stepwise selection of immune-related genes to identify a 12-gene signature with profound prognostic potential for CRC. Spearman transition model was originally constructed to quantify the level of cooperativity disorientation associated with progression from normal to precancerous to cancer tissue, which hopefully was able to reveal prognostic information that would probably have been missed by simply comparing gene expression levels between distinct sample types. Further investigation is needed to unravel the underlying molecular mechanism of these 12 genes, which may have diagnostic and therapeutic potential.

## Supporting Information

S1 Methods Spearman correlation transition model. (DOCX)

S1 Table Reactome enrichment of differentially expressed genes (DEGs) in colorectal cancer. (DOCX)

S2 Table Gene lists of the miRNA-mRNA regulatory network constructed using bioinformatics analysis and 60 paired mRNA and microRNA profiles from CRCs. (DOCX)

S3 Table KEGG pathways containing at least one gene of the 12-gene signature. (XLSX)
