# Supplementary material for: Discovery of a Novel Immune Gene Signature with Profound Prognostic Value in Colorectal Cancer: A Model of Cooperativity Disorientation Created in the Process from Development to Cancer
Source: PLoS One. 2015 Sep 1;10(9):e0137171. doi: 10.1371/journal.pone.0137171 (PMC4556644; doi:10.1371/journal.pone.0137171)
Supplement: S1 Methods — (DOCX) [file pone.0137171.s001.docx]

**S1 Methods. Spearman correlation transition model.**

Three general stages could be used to recapitulate the fate of human colorectal tissue from birth to death, i.e. embryonic development, precancerous progression, and cancer. Embryonic development is a highly organized biological process, and the correlations between immune genes should show a relatively compact and synchronized pattern to assure a safe and sound developmental process, which probably was severely disrupted (cooperativity disorientation) in precancerous and caner stage of colorectal cancer (CRC). Spearman transition model we originally proposed here made a preliminary endeavor to find the culprit genes responsible for disrupting this organized correlation pattern between immune genes during carcinogenesis.

Pair-wise Pearson correlation tests were accomplished among N development varying immune genes (DVIGs, suppose the number of DVIGs was N) in three stages. Suppose there are 2 genes A and B, if the correlation value between A and B is close to 1 or -1, the expressions of A and B are strongly synchronized; if the value is close to 0, the expression of A is random to B, and the chance of A and B having a biological association is relatively slim. Pearson correlation values were adjusted into with correlation test *p* value to eliminate the bias caused by different sample sizes using the formula:

The heat maps of development, progression and cancer were established based on corresponding N * N adjusted Pearson correlation values. First, in each stage, unsupervised clustering algorithm (UCA) was conducted to cluster DVIGs, rendering three diagonally symmetrical heat maps. Then, genes in progression and cancer heat maps were reordered in the same way as in the development stage. For a given DVIG, in each heat map, the values against all DVIGs (including itself) constitute an N-element vector designated as development intra-immune vector (DIV), progression intra-immune vector (PIV) and cancer intra-immune vector (CIV), respectively. The correlation value against itself (equal to 1, containing no information for correlation pattern disruption) in DIV, PIV and CIV was treated as missing value and imputed as the mean of its two neighbors (neighboring two element values in three reordered intra-immune vectors for each gene, respectively). For example, an intra-immune vector of a given gene is *X*_1_, *X*_2_,…*X*_k-1_, *X*_k_ , *X*_k+1_,… *X*_664_, *X*_665_, and *X*_k_ = 1 (correlation against itself). Then *X*_k_ is treated as a missing value, and *X*_k_ = (*X*_k-1_+ *X*_k+1_)/2. If *X*_1_ = 1, then *X*_1_ = *X*_2_; and if *X*_665_ = 1, then *X*_665_ = *X*_664_.

For a given gene, the corresponding intra-immune vector measured the biological association between this gene and any other immune gene. Thus, during the transition from embryonic development to precancerous progression to cancer, the genes with drastic rank order reshuffling (compromising intra-immune cooperativity, i.e. cooperativity disorientation) are probably responsible for cancer initiation or escalation.

In this regard, Spearman transition model was originally designed to nail down the genes accounting for cooperativity disorientation during carcinogenesis. Spearman transition between development and progression (STD-P) was the Spearman correlation between DIV and PIV, and that between progression and cancer (STP-C) was the Spearman correlation between PIV and CIV. STD-P numerically denoted the correlation pattern disruption during the transition between embryonic development and cancer initiation, and STP-C was to quantify the correlation pattern disruption during the transition between cancer initiation and cancer deterioration. Suppose there is a DVIG named A, whose STD-P (Spearman correlation between A’s DIV and PIV) is close to 1, indicating in the transition between development and progression stage, the rank order of Gene A’s closeness with other immune genes was nearly the same; STD-P is close to -1, meaning the rank order was nearly turned upside down; STD-P is close to 0, suggesting the rank order was randomly reshuffled. Likewise, STP-C of Gene A is the parameter denoting the transition of intra-immune cooperation between progression and cancer stage. Therefore, Spearman transition system was established by projecting each of N DVIGs onto a rectangular coordinate system with its corresponding STD-P and STP-C as x-axis and y-axis coordinate.

Point (1, 1) was defined as theoretically stable point (TSP). Suppose a gene was projected onto this TSP, it inferred the cooperativity status of this gene’s biological association with other immune genes in the whole process of carcinogenesis was absolutely stable. The closer to TSP a given gene is located in Spearman correlation transition system, the more stable of this gene’s cooperativity during carcinogenesis. Using this point as circle center, we drew a quarter of circle arc with the radius of 1 Euclidean distance (use 1 Euclidean distance as threshold), and genes within this circle arc (genes which did not contribute much to cooperativity disorientation) were discarded for further narrow-down procedures.
