# Supplementary material for: Discovery of a Novel Immune Gene Signature with Profound Prognostic Value in Colorectal Cancer: A Model of Cooperativity Disorientation Created in the Process from Development to Cancer
Source: PLoS One. 2015 Sep 1;10(9):e0137171. doi: 10.1371/journal.pone.0137171 (PMC4556644; doi:10.1371/journal.pone.0137171)
Supplement: S1 Table — (DOCX) [file pone.0137171.s002.docx]

**S1 Table. Reactome enrichment of differentially expressed genes (DEGs) in colorectal cancer.**

| **Term** | **Count** | **Percentage (%)** | ***P* Value** | **Fold Enrichment** | **Bonferroni** |
| --- | --- | --- | --- | --- | --- |
| REACT_152:Cell Cycle, Mitotic | 131 | 2.308 | 1.70E-05 | 1.353 | **0.001** |
| REACT_6900:Signaling in Immune system | 122 | 2.149 | 6.20E-05 | 1.34 | **0.004** |
| REACT_604:Hemostasis | 93 | 1.638 | 0.008 | 1.243 | 0.449 |
| REACT_1505:Integration of energy metabolism | 86 | 1.515 | 0.014 | 1.233 | 0.638 |
| REACT_13433:Biological oxidations | 48 | 0.846 | 0.027 | 1.3 | 0.865 |
| REACT_13552:Integrin cell surface interactions | 34 | 0.599 | 0.054 | 1.318 | 0.982 |
| REACT_383:DNA Replication | 39 | 0.687 | 0.073 | 1.263 | 0.996 |
| REACT_15295:Opioid Signalling | 33 | 0.581 | 0.073 | 1.295 | 0.996 |
