# Supplementary material for: Discovery of a Novel Immune Gene Signature with Profound Prognostic Value in Colorectal Cancer: A Model of Cooperativity Disorientation Created in the Process from Development to Cancer
Source: PLoS One. 2015 Sep 1;10(9):e0137171. doi: 10.1371/journal.pone.0137171 (PMC4556644; doi:10.1371/journal.pone.0137171)
Supplement: S2 Table — (DOCX) [file pone.0137171.s003.docx]

**S2 Table. Gene lists of the miRNA-mRNA regulatory network constructed using bioinformatics analysis and 60 paired mRNA and microRNA profiles from CRCs.**

| \| ***MiRNA name*** \| ***Predicted targets*** \| \| --- \| --- \| \| *hsa-miR-125b* \| *ABR RPS6KA1 TNFAIP3 LIF* \| \| *hsa-miR-34a* \| *AXL NOTCH2 PRKD1 SIRT1 PRKCE* \| \| *hsa-let-7e* \| *BCL2L1 IKBKE CHUK* \| \| *hsa-miR-19b* \| *BCL3 MAP2K3* \| \| *hsa-miR-19a* \| *BCL3 MAP2K3* \| \| *hsa-miR-27a* \| *CNR1 GATA3 CADM1 COLEC12 NCAM1* \| \| *hsa-miR-23a* \| *CXCL12 ZEB1 CADM1 ETS1 CNR1 MEF2A MAP3K1 JAM3 PNMA1 TGFB2 PXDN* \| \| *hsa-miR-200b* \| *EP300 ETS1 GEM ZEB1 NCAM1 FYN FOXF1* \| \| *hsa-miR-200c* \| *EP300 ETS1 GEM PIN1 ZEB1 NCAM1 FYN SIKE1 RPS6KA2 FOXF1* \| \| *hsa-miR-22* \| *EP300 SIRT1 TYRO3 MAPK10 EDA* \| \| *hsa-miR-20a* \| *ITCH MAP3K5 RPS6KA1 IRAK4* \| \| *hsa-miR-20b* \| *ITCH MAP3K5 RPS6KA1 CASP8 CHUK IRAK4 VASP* \| \| *hsa-miR-106b* \| *MAP3K5 RPS6KA1 LIF IRAK4 JAK1 VASP* \| \| *hsa-miR-93* \| *MAP3K5 RPS6KA1 IRAK4 VASP* \| \| *hsa-miR-30c* \| *MAP3K5 YWHAZ TNIP1 LYN* \| \| *hsa-miR-15b* \| *MYB* \| \| *hsa-miR-107* \| *MYB RPS6KA3 VAV3 TNFAIP3* \| \| *hsa-miR-130b* \| *MYB* \| \| *hsa-miR-130a* \| *MYB* \| \| *hsa-miR-25* \| *PTGER4* \| \| *hsa-miR-145* \| *YTHDF2 BCR AKIRIN2* \| \| *hsa-miR-214* \| *YWHAZ PVRL1* \| \| *hsa-miR-26a* \| *SRPK1* \| \| *hsa-miR-103* \| *MYB* \| \| *hsa-miR-17* \| *ITCH IRAK4 RPS6KA1 VASP MAP3K5* \| \| *hsa-miR-21* \| *PCBP2 MAPK10 TGFB2 CADM1 PRKCE* \| \| *hsa-miR-30a* \| *TNIP1 LYN JAK1* \| \| *hsa-miR-92a* \| *PTGER4* \| \| *hsa-miR-148a* \| *CNR1 CADM1 FOXF1* \| \| *hsa-miR-181a* \| *LIF* \| \| *hsa-miR-223* \| *CBLB SNCA ZEB1 PRKCE* \| \| *hsa-miR-140-5p* \| *BCL2L1* \| \| *hsa-miR-125a-5p* \| *RPS6KA1* \| \| *hsa-miR-301a* \| *MYB* \| \| *hsa-miR-342-3p* \| *BCL2L1* \| \| *hsa-miR-425* \| *NCAM1* \| \| *hsa-miR-320b* \| *YWHAZ* \| |  |
| --- | --- | --- | --- | --- | --- | --- | --- | --- | --- | --- | --- | --- | --- | --- | --- | --- | --- | --- | --- | --- | --- | --- | --- | --- | --- | --- | --- | --- | --- | --- | --- | --- | --- | --- | --- | --- | --- | --- | --- | --- | --- | --- | --- | --- | --- | --- | --- | --- | --- | --- | --- | --- | --- | --- | --- | --- | --- | --- | --- | --- | --- | --- | --- | --- | --- | --- | --- | --- | --- | --- | --- | --- | --- | --- | --- | --- | --- |
